# Contribution of the STAT Family of Transcription Factors to the Expression of the Serotonin 2B (HTR2B) Receptor in Human Uveal Melanoma

**DOI:** 10.3390/ijms23031564

**Published:** 2022-01-29

**Authors:** Manel Benhassine, Gaëtan Le-Bel, Sylvain L. Guérin

**Affiliations:** 1 Centre Universitaire d’Ophtalmologie-Recherche (CUO-Recherche), Axe Médecine Régénératrice, Hôpital du Saint-Sacrement, Centre de Recherche FRQS du CHU de Québec, Quebec City, QC G1S4L8, Canada; manal.benhassine.1@ulaval.ca (M.B.); gaetan.lebel17@gmail.com (G.L.-B.); 2 Département d’Ophtalmologie, Faculté de Médecine, Université Laval, Quebec City, QC G1V0A6, Canada

**Keywords:** HTR2B, STAT proteins, uveal melanoma, promoter, gene transcription

## Abstract

Uveal melanoma (UM) remains the most common intraocular malignancy among diseases affecting the adult eye. The primary tumor disseminates to the liver in half of patients and leads to a 6 to 12-month survival rate, making UM a particularly aggressive type of cancer. Genomic analyses have led to the development of gene-expression profiles that can efficiently predict metastatic progression. Among these genes, that encoding the serotonin receptor 2B (HTR2B) represents the most discriminant from this molecular signature, its aberrant expression being the hallmark of UM metastatic progression. Recent evidence suggests that expression of HTR2B might be regulated through the Janus kinase/Signal Transducer and Activator of Transcription proteins (JAK/STAT) intracellular signalization pathway. However, little is actually known about the molecular mechanisms involved in the abnormally elevated expression of the *HTR2B* gene in metastatic UM and whether activated STAT proteins participates to this mechanism. In this study, we determined the pattern of STAT family members expressed in both primary tumors and UM cell-lines, and evaluated their contribution to *HTR2B* gene expression. Examination of the *HTR2B* promoter sequence revealed the presence of a STAT putative target site (5′-TTC (N)3 GAA3′) located 280 bp upstream of the mRNA start site that is completely identical to the high affinity binding site recognized by these TFs. Gene profiling on microarrays provided evidence that metastatic UM cell lines with high levels of HTR2B also express high levels of STAT proteins whereas low levels of these TFs are observed in non-metastatic UM cells with low levels of HTR2B, suggesting that STAT proteins contribute to *HTR2B* gene expression in UM cells. All UM cell lines tested were found to express their own pattern of STAT proteins in Western blot analyses. Furthermore, T142 and T143 UM cells responded to interleukins IL-4 and IL-6 by increasing the phosphorylation status of STAT1. Most of all, expression of HTR2B also considerably increased in response to both IL-4 and IL-6 therefore providing evidence that *HTR2B* gene expression is modulated by STAT proteins in UM cells. The binding of STAT proteins to the −280 HTR2B/STAT site was also demonstrated by electrophoretic mobility shift assay (EMSA) analyses and site-directed mutation of that STAT site also abolished both IL-4 and IL-6 responsiveness in in vitro transfection analyses. The results of this study therefore demonstrate that members from the STAT family of TFs positively contribute to the expression of HTR2B in uveal melanoma.

## 1. Introduction

Uveal melanoma (UM) is the most common intraocular malignancy in adults, with an incidence of four to six affected individuals per million in the United States [1]. Despite effective primary therapy, approximately 50% of patients will develop the metastatic disease [2]. Liver metastasis is a dreaded complication of this cancer as patients rarely survive more than five years following the initial detection of metastasis, with a death rate reaching 92% at two years [3]. Microarray analyses identified 12 genes, designated as the UM gene expression signature, that can distinguish between UM primary tumors that are at low or high risk of progressing towards the liver metastatic disease. The human gene encoding the 5-Hydroxytryptamine receptor 2B (*HTR2B*), also known as the serotonin receptor 2B, turns out to be the most discriminating among the class II genes for the identification of UM patients at high risk of evolving toward formation of liver metastases [4,5,6]. The serotonin receptors to which HTR2B belongs are gathered into a family of proteins that can be divided in seven subfamilies (HTR1-7). Interestingly, HTR2B has been described as an oncogene in hepatocellular carcinoma (HCC), prostate, small intestine and breast cancers [7,8,9], but as a tumor suppressor in ovarian cancer [10]. In addition to its function as a neurotransmitter, serotonin plays a role in its development, and most of its biological actions are transmitted within the cell through the activation of a few signal transduction pathways including the phospholipase C (PLC), the Receptor Tyrosin Kinase (RTK)/Phosphatidylinositol-4,5-bisphosphate-3-kinase (PI3K)/Extracellular signal-Regulated Kinase (ERK)/mammalian target of rapamycin (mTOR), the RAF/Mitogen activated protein Kinase Kinase (MEK)/ERK and the Janus kinase/Signal Transducer and Activator of Transcription proteins (JAK/STAT) pathways [10,11,12,13,14,15]. Although the serotonin-mediated activation of the JAK/STAT pathway has been shown to rely essentially on the 5-HT1A or 5-HT2A receptors [14,16,17,18], recent evidence also suggests that the HTR2B receptor might participate as well in the activation of this signal transduction pathway in uveal melanoma [19].

STAT proteins have the ability to transduce signals from the cell membrane into the nucleus, where they can alter the transcription of many responsive genes. Today, seven STAT genes have been identified in the human genome: *STAT1* to *STAT4*, *STAT5a*, *STAT5b* and *STAT6* [20]. STAT signaling is involved in many normal physiologic cell processes, including proliferation, differentiation, angiogenesis, immune system regulation and apoptosis. However, aberrant STAT regulation that may result from many possible irregularities can lead to various pathologic events, such as malignant cell transformation and metastasis [21]. It is noteworthy that some STAT proteins are currently even considered as oncogenes [22]. The aberrant activation of STAT proteins, most particularly STAT1, STAT3 and STAT5, has been suspected or proposed to significantly contribute to the progression of a variety of human tumors and cancer cell lines, including hematologic malignancies and solid tumors (reviewed in [23]).

Until very recently, STAT3 and STAT5 were probably the STAT family members with the most significant role in cancer development, in particular STAT3, as it clearly turned out to be the most thoroughly investigated STAT protein in cancer research. However, studies that relate cancer development to aberrant activation of STAT1 have blown-up over the last few years, the abnormal expression of STAT1 being now even used as a prognostic factor for patients with solid tumors [24]. By its capacity to control the immune system and promote tumor immune surveillance, STAT1 has been recognized as a tumor suppressor in breast cancer [25]. STAT2, STAT4 and STAT6 appear to have more limited roles in tumor biology. STAT proteins therefore play a major regulatory role in the maintenance and survival of cancer cells by allowing them to escape the host’s anti-tumor responses. Cancer cells can then expand, metastasize and progress toward formation of solid tumors.

All STAT proteins have been reported to bind as dimers with varying affinities to the same palindromic DNA regulatory element (DRE: 5′-TTCN_2–4_GAA-3′) that is present in both hormone- and cytokine-responsive genes ([26]; also reviewed by [20,27]). Once they recognize promoter proximal DREs, STAT proteins then alter either positively (activation) or negatively (repression) the transcription of their target genes. STATs can also control enhancer activity, epigenetic status of associated genes, or instruct non-coding loci (e.g., miRNAs) by engaging more distal binding DREs.

The intercellular communication regulating developmental signaling is precisely controlled by both regulatory feedback loops that protect cells against signals that may be produced in the wrong time or place and which may lead to inappropriate developmental responses such as is frequently happening in cancer tissues. Such feedback loops can either be positive or negative depending on whether a gene increases or represses its own expression, respectively. It is believed that the most obvious use of negative feedback is to limit the duration of a signal that induces its own negative regulator so that when a threshold has been reached, this signal disappears. Cytokine signaling through the JAK/STAT signaling pathway well illustrates this mechanism. As many hormones, cytokines and growth factors receptors transduce their signals through this pathway, it is not surprising that regulatory feedback loops control their expression. For instance, expression of the glucocorticoid receptor (GR) has been shown to be under the regulatory influence of a negative feedback loop involving miR-29a [28]. Similarly, a negative feedback loop involving the transcription factor GATA2 also controls the transcription of the androgen receptor (ER) upon androgen deprivation in castration-resistant prostate cancers [29]. On the other hand, expression of the estrogen receptor (ER) has been demonstrated to be under the control of a positive feedback loop involving IL6 in oesophageal squamous cell carcinoma [30]. Therefore, in many instances, cancer cells appear to escape the cell’s surveillance mechanism by altering the function of cellular targets within these regulatory feedback loops that are primed to respond to such signals.

Although studies that evaluated the relationship of STAT family members with cancer progression are particularly abundant, only a few examined activation of STAT proteins by serotonin in uveal melanoma [19,31]. In vascular smooth muscle cells, serotonin was found to activate JAK1, JAK2, and STAT1 through the HTR2A receptor [14]. However, blocking the signal transduction cascade mediated by the binding of serotonin to the HTR2B receptor using the selective serotonin receptor 2B antagonist PRX-08066 considerably reduced the phosphorylation of STAT2, STAT3, STAT6, and most remarkably that of both STAT5A and STAT5B, in three different UM cell lines [19] therefore demonstrating that HTR2B ligand recognition activates STAT proteins in UM. In the present study, we demonstrated that UM tumors and their derivative cell lines express different combinations of STAT genes and proteins. In addition, the *HTR2B* gene promoter was shown to bear multiple putative target sites for STAT proteins of which one (located at position −280 relative to the *HTR2B* mRNA start site) functionally binds members of the STAT family. Most of all, expression of HTR2B was found to positively respond to the STAT protein inducers IL-4 and IL-6, a mechanism ensured at least in part through the −280 STAT DRE, thereby further supporting the existence of an interleukin/JAK/STAT signalization pathway that may contribute to the metastatic properties of uveal melanoma.

## 2. Results

### 2.1. Multiple Putative DNA Target Sites for STAT Proteins Are Present in the Human HTR2B Gene Promoter and 5′-Flanking Sequences

In order to verify whether transcription of the *HTR2B* gene might be under the regulatory influence of STAT family members, we first searched for the presence of putative STAT DREs in both the promoter and 5′-flanking sequence of the *HTR2B* gene. A large segment of that gene extending up to approximately 2 Kbp upstream from the *HTR2B* mRNA start site was subjected to a search with the TFSEARCH program, a tool for the identification of putative DNA target sequences for nuclear-located transcription factors. We searched for putative STAT target sites that deviate from the consensus STAT DRE (5′-TTCN2–4GAA-3′) by no more than one nucleotide. Thirteen STAT target sites that fitted into this category could be identified along the entire promoter and 5′-flanking sequence of the *HTR2B* gene (Figure S1). Among them, three DREs that perfectly match the STAT consensus sequence could be identified at positions −982, −942 and −280 relative to the *HTR2B* mRNA start site (Figure S1B). The STAT DRE located at position −280 sounded particularly interesting in that most STAT proteins (especially STAT1 and STAT5) have an increased affinity for DREs in which both halves of the palindromic sequence are separated by a 3 nucleotides linker (as opposed to 2 and 4 nucleotides for the −982 and −942 DREs, respectively) [26]. On the other hand, STAT6 appears to clearly prefer a linker sequence comprising 4 nucleotides, as observed for the −942 DRE [26]. Therefore, we can conclude that multiple putative STAT binding sites are present in both the 5′-flanking and promoter sequence of the *HTR2B* gene.

### 2.2. Expression of STAT Genes Correlates with That of HTR2B in Uveal Melanoma

**Figure 1:**
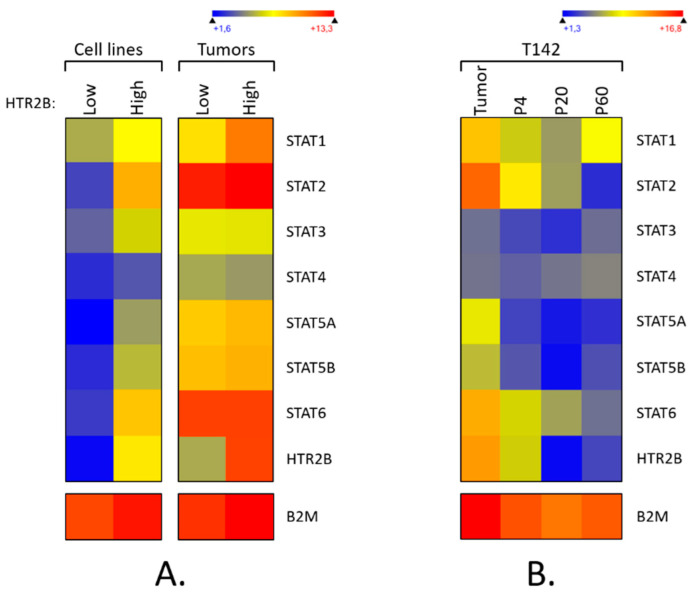
Expression of STAT genes in uveal melanoma. (**A**) Heatmap representation of all the STAT genes expressed by UM cell lines or the primary tumors from which they have been cultured. Data show the average of the individual transcriptome profile extracted from UM cell lines that express either low (T97, T108, T111, T128, T131, T132 and T143 cell lines) or high levels of *HTR2B* (T98, T142, T151 and T157 cell lines), or from primary tumors with either low (tumors 140, 149, 154 and 157) or high *HTR2B* levels (tumors 138, 139, 141, 142, 147 and 151). A dark blue color corresponds to a very low level of expression, whereas high levels appear in yellow/red. Moderate to high levels of all STAT genes except *STAT4* are observed in UM cell lines with high levels of *HTR2B* but not in UM cells with low expression of *HTR2B*. In UM primary tumors, only *STAT1* expression is considerably reduced in tumors with low *HTR2B* levels. (**B**) Heatmap representation of all STAT genes expressed by the UM primary tumor 142 (tumor) and its derivative cell line T142 cultured at passages P4, P20 and P60 as determined by DNA microarrays. As for *HTR2B*, expression of STAT genes, which is elevated in the primary tumor 142, is rapidly lost with cell passaging in the derivative T142 UM cell-line.

We have previously shown that the expression of the *HTR2B* gene at the transcriptional level correlates with the aggressiveness of the primary tumors from which UM cell lines are cultured [32]. We therefore searched our gene profiling on microarray datafiles to sort out which of the STAT genes are expressed by our UM cell lines and primary tumors, and attempted to correlate STAT genes expression with that of the *HTR2B* gene. Gene profiling analyses were conducted using total RNAs extracted from UM cell lines that express either low (T97, T108, T111, T128, T131, T132 and T143 cell lines) or high levels of *HTR2B* (T98, T142, T151 and T157 cell lines), or from primary tumors with either low (140, 149, 154 and 157 tumors) or high *HTR2B* levels (138, 139, 141, 142, 147 and 151 tumors) and used for microarray analyses. As shown on Figure 1A (and Figure S2), both UM cell lines (left) and primary tumors (right) that express high levels of *HTR2B* also express moderate to high levels of all STAT genes, with the only exception being *STAT4* (pooled data are presented; data for each individual cell line and tumor are shown on Supplementary Figure S1). On the other hand, a dramatic reduction in the expression of all STAT genes is observed in UM cell lines that have low *HTR2B* levels, whereas only *STAT1* expression is considerably reduced in low HTR2B tumors. Therefore, expression of *HTR2B* correlates perfectly with that of all STAT genes in UM cell lines whereas it is coordinated with that of STAT1 in the UM primary tumors.

We recently reported that cell passaging severely reduces the expression of genes encoding markers typical of UM, including those of the prognostic gene signature such as *HTR2B* [33]. We therefore examined whether the cell passage-dependent reduction in the expression of *HTR2B* also correlates with similar decreases in the expression of the STAT genes in T142 UM cells when compared to its primary tumor. As for *HTR2B*, expression of *STAT1*, *STAT2*, *STAT5A*, *STAT5B*, and *STAT6* genes, which were moderately to highly expressed in the primary tumor 142, was rapidly lost with cell passaging in the derivative T142 UM cell line (Figure 1B; similar results were also obtained with the 143 primary tumor and its derivative cell line T143 (data not shown)). These results suggest that expression of *HTR2B* correlates with that of STAT genes at the transcriptional level in uveal melanoma.

**Figure 2:**
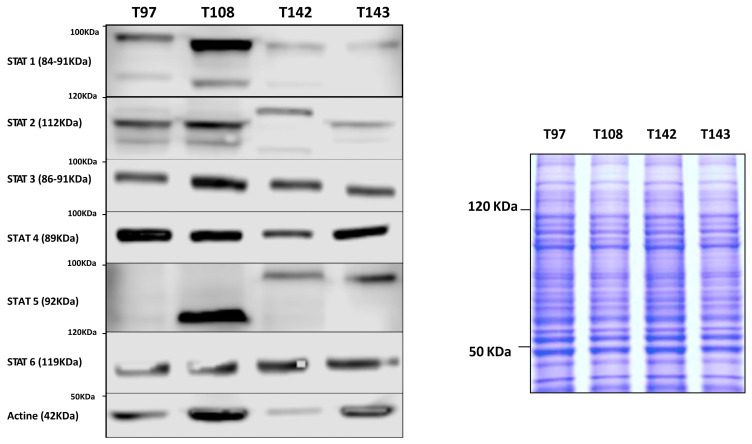
Western blot analysis of the STAT isoforms in UM cells. Total proteins isolated from the UM cell lines T97, T108, T142 and T143 were Western blotted using polyclonal antibodies directed against all STAT isoforms (STAT1 to STAT6). Actin was also blotted as a control. Coomassie blue staining (25 μg of each protein extract was used) is also shown beside the Western blots as a protein loading control. UM cell lines were found to express all STAT proteins to varying levels. In addition, the metastatic T142 cell line expresses a STAT2 isoform with an apparent molecular mass higher than that observed in the remaining UM cells.

We next examined the pattern of STAT proteins expressed in UM cell lines cultured either from metastatic (T142) or non-metastatic (T97, T108 and T143) UM primary tumors. As shown in Figure 2, all STAT proteins are expressed at varying levels by all UM cell lines, irrespective of whether they have been cultured from metastatic tumors or not. 

Data are also presented for the housekeeping gene β-2-microglobulin (B2M; control). Whereas STAT2, STAT3, STAT4 and STAT6 are more uniformly expressed between all four UM cell lines, high levels of STAT1 expression are observed in T108 cells, T97, T142 and T143 cells expressing, on the other hand, low to very low levels of this isoform. Interestingly, T97 apparently expresses no STAT5 whereas this isoform has a faster electrophoretic mobility in T108 UM cells. Furthermore, the metastatic T142 cell line expresses a STAT2 isoform with an apparent molecular mass higher than that observed in the remaining UM cells.

### 2.3. Expression of HTR2B Responds to Stimulation by IL4 and IL6 in Uveal Melanoma

**Figure 3:**
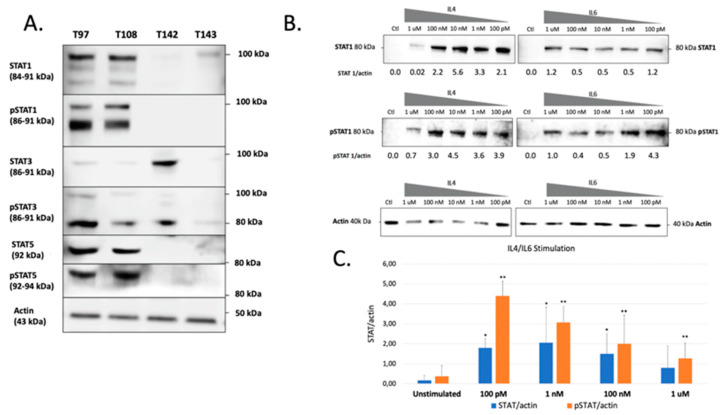
Western blot analysis of total and phosphorylated STAT1, STAT3 and STAT5 in UM cell lines. (**A**) Proteins from the UM cell lines T97, T108, T142 and T143 were Western blotted using antibodies that recognize only the phosphorylated (phospho-STAT1, phosphor-STAT3 and phosphor-STAT5) or total (comprising both inactive and phosphorylated) STAT1, STAT3 or STAT5 proteins (STAT1, STAT3 and STAT5). T97 and T108 UM cells express phosphorylated STAT1, STAT3 and STAT5 whereas T142 and T143 only express moderate and low levels of phospho-STAT3, respectively. The molecular mass of each STAT isoform is indicated in parenthesis. Actin is shown as a control. (**B**) Western blot analysis of either total (STAT1) or phosphorylated (pSTAT1) STAT1 in T142 UM cells that have been grown alone (Ctl) or in the presence of increasing doses (100 pM to 1 µM) of IL-4 and IL-6. The ratio of total (STAT1/actin) and phosphorylated (pSTAT1/actin) STAT1 over that of actin is also shown for both IL-4 and IL-6 stimulation. (**C**) Graph representation of the STAT/actin and pSTAT/actin ratios in either unstimulated or IL-4/IL-6 stimulated T142 UM cells. The addition of either IL-4 or IL-6 dramatically increased expression of total STAT1. Actin is shown as a control. * and **: Values considered to be statistically significant from those obtained for unstimulated total (STAT/actin) and phosphorylated (pSTAT/actin) STAT1, respectively (*p* value < 0.01).

The previous experiments demonstrated that all UM cells express STAT proteins to different levels. However, this does not necessarily reflect the activation status of these transcription factors in unstimulated cells. We therefore verified both the total and phosphorylated STAT1, STAT3 and STAT5 proteins present in all our UM cell lines. These STAT family members were selected for this analysis as they are the most often involved in cancer development and also because they are expressed to relatively high levels in UM cells. As shown on Figure 3A, a large proportion of the STAT1, STAT3 and STAT5 proteins expressed by T97 and T108 is phosphorylated. On the other hand, T142 also expresses phospho-STAT3 but has no detectable phospho-STAT1 or phospho-STAT5. As for T142, T143 UM cells also had no detectable phospho-STAT1 nor phospho-STAT5 and only a weak level of phospho-STAT3. As T142 has a barely detectable level of total STAT1 and no activated STAT1, we therefore selected this UM cell line to verify whether it would respond to stimulation of STAT1 activation by interleukins 4 (IL-4) and 6 (IL-6). As expected, control T142 UM cells expressed no detectable level of total or phosphorylated STAT1 (Ctl; Figure 3B). However, the addition of either IL-4 or IL-6 dramatically increased expression of total STAT1 (Figure 3B,C). Furthermore, both these interleukins proved to be very efficient at activating STAT1, since as little as 100 pM considerably increased phosphorylation of this transcription factor in T142 cells relative to the unstimulated controls (Figure 3B).

**Figure 4:**
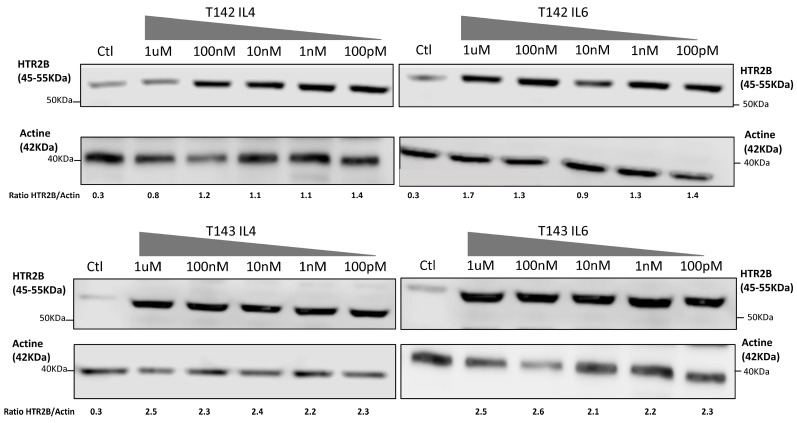
Expression of HTR2B in response to stimulation with IL-4 and IL-6. Expression of HTR2B was monitored by Western blot in both T142 and T143 UM cells cultured either alone (Ctl) or in the presence of increasing concentrations of IL4 and IL6 (100 pM to 1 µM). Values shown beneath each blot correspond to the ratio of the HTR2B signal over that of actin. Both IL-4 and IL-6 considerably increased expression of HTR2B in T142 and T143 UM cells relative to untreated cells (negative controls).

That three DREs with a perfect match to the STAT consensus sequence could be found in the HTR2B gene promoter and 5′-flanking sequence does not warrant that they would resolve the appropriate activated STAT response in vitro. In order to verify whether expression of HTR2B indeed responds to activated STAT proteins, we cultured both T142 and T143 UM cells in the presence of varying concentrations (100 pM to 1 µM) of IL-4 and IL-6 and evaluated whether this would alter expression of HTR2B at the protein level. Consistent with the results shown on Figure 3, expression of HTR2B considerably increased in both T142 (4.7-fold increases in the HTR2B/actin ratio in the presence of IL-4 and IL-6 relative to negative controls, respectively) and T143 UM cells (7.7- and 11.5-fold increases in the HTR2B/actin ratio in the presence of IL-4 and IL-6 relative to controls, respectively) when as little as 100 pM of either IL-4 and IL-6 were added to the culture medium, relative to untreated cells (negative controls) (Figure 4). The HTR2B/actin ratio remained fairly stable as the concentration of both IL-4 and IL-6 is increased to 1 µM. We therefore conclude that expression of HTR2B increases in response to STAT-mediated interleukin signaling.

### 2.4. The −280 STAT Target Site Binds STAT Proteins and Contributes to HTR2B Promoter Activity In Vitro

**Figure 5:**
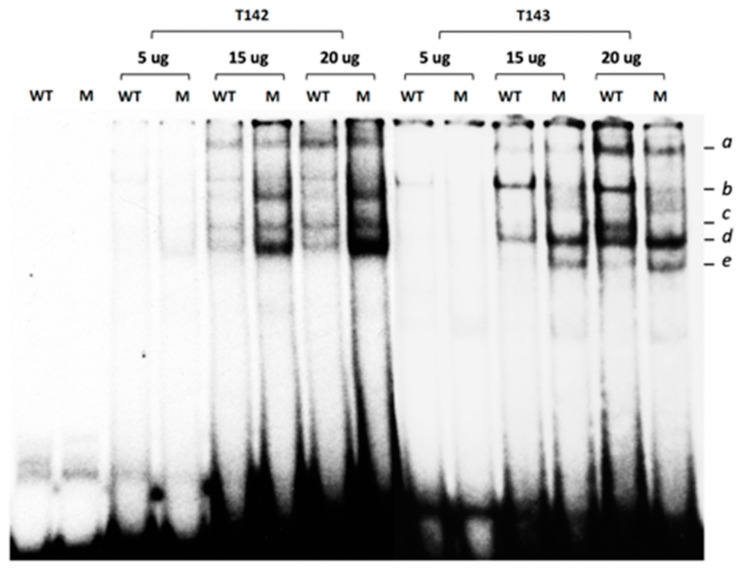
Gel shift analysis of the nuclear proteins binding to the *HTR2B* −280 STAT site. Nuclear proteins (5-, 15- or 20 µg) obtained from the UM cell lines T142 and T143 were incubated with a 5′ end-labeled, double-stranded oligonucleotide bearing either the wild-type sequence of the −280 STAT site identified in the *HTR2B* gene promoter (WT), or a derivative in which the −280 STAT site was mutated (M). Formation of DNA-protein complexes was then monitored by EMSA. The position of the multiple DNA-protein complexes formed is indicated (*a* to *e*), along with that of the free probe (U). Incubation of increasing concentrations of T142 and T143 nuclear proteins with the WT −280 STAT labeled probe yielded the formation of distinct DNA-protein complexes of which only complex *b* completely disappeared when the wild-type probe was substituted by the mutated −280 STAT site (Mut).

As stated earlier, three distinct DNA regulatory elements that perfectly match the prototypical STAT target site have been identified in the HTR2B gene promoter (Supplementary Figure S1B). Because the −280 STAT DRE has its palyndromic repeats separated by a three nucleotides linker, a configuration that is also the most preferred for DNA binding of most STAT isoforms [26], it was selected to conduct the experiments in an electrophoretic mobility shift assay (EMSA). Therefore, 29 bp, double-stranded synthetic oligonucleotides bearing either the wild-type DNA sequence of the HTR2B −280 STAT site (WT), or a mutated STAT derivative in which the T, T and G residues at positions −284, −283 and −178 were changed for A, A and G, respectively (Table S1), were [^32^P] 5′-end-labelled and used as labeled-probes in EMSAs. Incubation of increasing concentrations (5-, 15- and 20 µg) of nuclear proteins prepared from both T142 and T143 UM cells with the WT −280 STAT labeled probe yielded the formation of five distinct DNA-protein complexes (a to e) on a native polyacrylamide gel (Figure 5). Interestingly, substituting the wild-type probe for the mutated −280 STAT site (Mut) led to the complete disappearance of complex b (this is particularly evident using the T143 proteins) when the same amount of extracts from both T142 and T143 cells were used as the source of proteins, suggesting that formation of complex b results from the recognition of the WT labeled probe by STAT proteins. Furthermore, we also observed an increase in the DNA binding of complex d when extracts from T142 and T143 UM cells were used, respectively (Figure 5).

**Figure 6:**
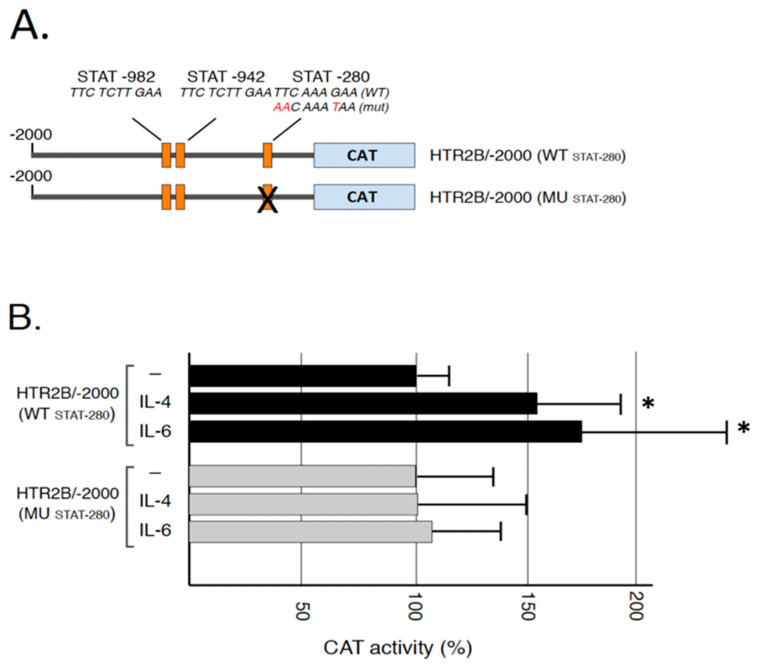
Responsiveness of the *HTR2B* gene promoter to IL-4 and IL-6 in transfected T108 cells: (**A**) Representation of the HTR2B/CAT recombinant plasmids used for transfection analyses. Numbers indicate the position relative to the *HTR2B* theoretical mRNA start site. The position of the three conserved STAT sites is indicated (STAT-982, STAT-942 and STAT-280) (**B**) CAT activities measured following transfection of the *HTR2B* constructs shown in panel B in the UM cell line T108. CAT activity is expressed relative to the level directed by the HTR2B/-2000 construct. *: Values considered to be statistically significant from those obtained with the HTR2B/-138 construct (*p* value < 0.01). Addition of IL-4 and IL-6 increased CAT activity encoded by the plasmid HTR2B/-2000(WT_STAT-280_) but not when the −280 STAT site is mutated in HTR2B/-2000(MU_STAT-280_).

We recently cloned different segments from the HTR2B gene promoter and 5′-flanking sequences upstream from the chloramphenicol acetyl transferase (CAT) reporter gene in order to characterize the regulatory elements modulating expression of that gene in UM cells. This study led to the demonstration that HTR2B gene transcription was down-regulated by the presence of both a proximal and distal silencer element that proved functional only in non-metastatic UM cells in vitro [32]. As the −280 STAT target site identified in the present study is located right in the middle of the proximal silencer, we examined whether both IL-4 and IL-6 can impact on the activity directed by the HTR2B gene promoter and whether mutation of this STAT site would alter IL-4 and IL-6 responsiveness in vitro. Therefore, the −280 STAT site was mutated in a version of the HTR2B gene that includes 2 kb of the 5′ promoter and flanking sequence (in the HTR2B/-2000 construct) (Figure 6A). Because they are much easier to transfect than any other of our UM cell lines, the plasmids bearing both the wild-type −280 STAT site and its mutated derivative were transfected in T108 UM cells and the CAT activities were determined. The addition of 100 nM IL-4 and IL-6 significantly increased by 54% and 67% the CAT activity directed by HTR2B/-2000, respectively (in HTR2B/-2000(WT_STAT-280_); Figure 6B). On the other hand, mutation of the −280 STAT site in HTR2B/-2000(MU_STAT-280_) entirely abolished both IL-4 and IL-6 responsiveness in transfected T108 UM cells.

## 3. Discussions

We recently investigated the molecular mechanisms that contribute to the increased expression of HTR2B in metastatic UM cells and demonstrated that both transcription factors Nuclear factor I (NFI) and Runt-related transcription factor 1 (RUNX1) interact with regulatory elements from the *HTR2B* gene to either activate (NFI) or repress (RUNX1) *HTR2B* expression in UM cells [32]. However, these TFs alone are insufficient to explain the elevated level of HTR2B protein observed in metastatic UM cells. The purpose of the present study was to investigate the potential contribution of proteins from the STAT transcription factors family to the expression of HTR2B in human UM cells. We demonstrated that all UM cell lines express their own combination of STAT isoforms and that some of them are obviously activated through phosphorylation. Furthermore, both IL-4 and IL-6 proved to be very efficient at activating STAT1 in metastatic T142 UM cells. Most of all, exposure to IL-4 and IL-6 dramatically increased the expression of HTR2B in both T142 and T143 UM cells. We could identify multiple putative target sites for STAT proteins within the promoter and 5′-flanking sequence of the *HTR2B* gene and demonstrated the binding of STAT proteins to the DRE located at position −280. Mutation of this −280 STAT DRE totally abolished responsiveness of the *HTR2B* basal promoter toward IL-4 and IL-6, therefore establishing that transcription of the *HTR2B* gene is under the regulatory influence of STAT proteins in uveal melanoma.

We recently reported that the 292bp *HTR2B* promoter segment extending from positions −138 to −430, and therefore comprising the −280 proximal STAT DRE, shares a negative regulatory function typical of silencer elements [32]. That same DNA area was also shown to bind members of the NFI family of transcription factors, although the positive regulatory influence they exert through binding of this NFI site (located at position −210) was rather weak [32]. Therefore, STAT proteins that interact with the −280 site are also located close to other transcription factors, such as NFI, that also bind nearby the −280 STAT site. Besides this promoter proximal negative regulatory region, a more distal silencer element (located from positions −1297 to −710) was also shown to bind the positive transcription factor RUNX1 [32]. Direct interaction between STAT and RUNX proteins that mutually inhibits their transcriptional activity has been reported [34]. Furthermore, NFI-B2, a member from the NFI family of TFs, and STAT5 were found to bind nearby DNA target sites in the promoter of the whey acidic protein (WAP) to regulate its expression during pregnancy in the mouse mammary gland, although no direct physical interaction between both factors was demonstrated [35,36]. The fact that mutations that also prevent binding of STATs to the −280 STAT site did not prevent the formation of other DNA-protein complexes in EMSA (for instance, that of complexes *a*, *d* and *e*; Figure 6A) also suggests the presence of additional transcription factor binding sites located nearby, or overlapping with the −280 STAT target site. That an increased signal is observed for some of these DNA-protein complexes (such as for complex *d*) when the −280 STAT DRE is mutated is consistent with the possibility that it overlaps with the target sites recognized by these yet unknown factors. Unlike with the non-metastatic T97, T108 and T143 UM cell lines, both the *HTR2B* proximal and distal silencers are inactive in T142 metastatic cells. Therefore, STAT proteins most likely compete with the negative regulatory transcription factors that ensure the functionality of these silencer elements and a delicate balance between them must dictate the level to which the *HTR2B* gene is transcribed in non-metastatic UM cells. In metastatic T142 cells, this delicate balance is apparently shifted toward a regulatory function primarily ensured by positive regulatory TFs, including STAT proteins.

The increase in the molecular mass of STAT2 (a slight increase in that of STAT5 was also observed) in the metastatic T142 UM cell line relative to its apparent *M*_W_ in the non-metastatic T143 cell line is believed to result from post-translational modifications (PTMs) in T142 cells that are not occurring in T143 UM cells. Besides PTMs such as phosphorylation, ubiquitination, ISGylation, SUMOylation and acetylation, that do not significantly affect the molecular mass of the affected proteins, STATs have also been shown to be subjected to glycosylation, which, on the other hand, can cause substantial alterations in the electrophoretic mobility of the targeted proteins [37,38]. Indeed, wheat germ agglutinin affinity chromatography revealed that STAT1, STAT3, STAT5A, STAT5B and STAT6 are glucose-modified through the addition of O-linked N-acetylglucosamine (O-GlcNAc) residues on threonine or serine residues [39]. Serine/threonine phosphorylation of STAT1, STAT3 and STAT5 has been shown to contribute to the etiology of certain human cancers and immunodeficiencies [40]. In many cancers, STAT5 activation and its oncogenic gene expression is not only enhanced, but also kept persistent, whereas signaling involving activation of STAT5 is rather transient under physiological conditions. Interestingly, cancer-specific metabolic changes enhance glycosylation, which subsequently modulates STAT5 activity through enhanced tyrosine phosphorylation. Reducing the glycosylation status of the hyper-phosphorylated STAT5A variant, via glucose depletion or hypoxia, has been reported to restore transcription of oncogenic target genes back to their wild type level [41]. Glycosylation of proteins at threonine and/or serine residues, including transcription factors such as Sp1, SMAD4, DeltaLf (Delta-lactoferrin) and Nrf1 (Nuclear factor E2-related factor 1), to name a few, has been suggested to protect them from proteasomal degradation by masking nearby amino acids that are normally ubiquitinated [42,43,44,45,46], therefore increasing their steady-state stability. Therefore, and based on these observations, we can assume that the glycosylation status of both STAT2 and STAT5 might be related to the aggressiveness of the T142 UM cell line by ensuring an abnormally elevated intracellular signalization which also causes an increased expression of their target genes, such as HTR2B. Further experiments aimed at investigating the glycosylation status of STAT proteins in UM cells will surely prove particularly interesting as it may link this PTM to the UM metastatic properties.

Besides the −280 STAT site, our search for putative STAT target sites in the *HTR2B* promoter also revealed the presence of two other sequences with a perfect match to the STAT consensus target site at positions −982 and −942 relative to the *HTR2B* mRNA start site. The fact that mutation of the −280 proximal STAT site entirely abolished interleukin responsiveness in the context of the HTR2B/-2000 suggests that neither of these distant STAT sites can rescue the IL-4 and IL-6 responsiveness in T108 cells when the −280 STAT site is mutated. However, the apparent lack of functionality for both of these STAT distant sites might be context-dependent and a more in-depth characterization will be required before one can assume that neither are contributing to *hTR2B* gene expression in uveal melanoma.

The fact that both IL4 and IL6 not only contributed to the activation of STAT1 in T142 UM cells but also somehow restored its expression at the protein level (as very little STAT1 protein could be detected by Western blot in unstimulated UM cells) is particularly interesting, as it suggests *STAT1* gene transcription to be under the control of a positive feedback loop in T142 cells. Indeed, STAT1 has been shown to contribute to the transcription of its own gene through the presence of multiple STAT binding sites located within the *STAT1* gene proximal promoter, and mutation of these sites was found to disrupt reporter gene activity in response to leukemia inhibitory factor (LIF) [47]. Both the presence of an immune inflammatory phenotype and the tumor size correlates with a poor clinical prognosis in uveal melanoma. Interestingly, abnormally elevated levels of many cytokines, including IL-2, IL-4, IL-6 and IL-8, have been observed in the vitreous of eyes from patients with uveal melanoma [48,49,50,51,52]. IL-6 appears to be an important player in UM tumor progression, as increased expression in the level of this cytokine also correlates with an increased tumor prominence and the presence of both macrophage and T_reg_ infiltration of the primary tumor [48]. Among the tumor hallmarks of UM angiogenesis, the IL6-JAK-STAT3 pathway has been well-described to promote cancer progression as well as immunosuppression in an autocrine manner [53]. In the UM, activation of this signalization cascade also induces the trans-activation of the JunB subunit from the transcription factor AP-1, which, in turn, also promotes UM epithelial-mesenchymal transition and aggressiveness in UM [54].

Systemic therapies, including immunotherapy, have yielded poor results in the treatment of uveal melanoma [55]. Therefore, searching for new immune modulatory targets, incoming immunotherapy biomarkers and combined immune strategies with drugs offer a new therapeutic paradigm. Recent studies have shown an encouraging result in cutaneous melanoma (phase I clinical study) using these approaches [55]. However, despite the common origin from neural crest-derived cells, uveal and cutaneous melanomas have few overlapping genetic signatures. As a consequence, many therapies that have proven effective in cutaneous melanoma have little or no success in uveal melanoma. Immunotherapy with checkpoint inhibition showed promising results in the treatment of cutaneous melanoma, however, it did not appear to be equally effective with uveal melanoma. Moreover, angiogenesis seems to confer a worse prognosis to UM when compared to cutaneous melanoma [56]. Better insight into the molecular and genetic profile of uveal melanoma, such as the interest given in our study to the contribution of STAT family members to the expression of the serotonin receptor HTR2B, will facilitate the identification of new prognostic biomarkers and thus enable us to adapt the existing immunotherapy procedures in order to develop new forms of treatments specifically designed for uveal melanoma patients [57]. STAT family members have been involved in human cancer progression, development, survival, and resistance to treatment. This is especially the case for both STAT3 and STAT5 that are considered either as oncogenes or tumor suppressors, depending on the context and the delicate balance between the different counteracting transcription factors involved [23]. Immunotherapy approaches have been extensively investigated in recent years, and since these transcription factors are key members in the immune system response, it comes as no surprise that they are also embedded in the growing collection of potential new immune modulatory targets. Assessing the STAT signaling pathway and expression of its constituting mediators have been shown to predict sensitivity to immunotherapy and targeted STAT inhibition [23]. Knowing that STAT members are under the control of immune, interleukin/cytokine signals that differ from one patient to another may prove particularly informative as to whether any specific patient is a potential candidate for immunotherapy, depending on his STAT/interleukins/cytokines expression status.

In immunotherapy approaches, most attention is paid to the targeted inhibition of immune checkpoints using monoclonal antibodies, especially against PD-1 (programmed cell death protein 1), a cell-surface receptor that acts to restrain T cell-mediated immune responses when activated by its specific ligand PD-L1 (programmed death ligand 1) [58]. STAT1 and STAT3 are considered as potential biomarkers to define patients who are more likely to respond to immunotherapy, as both these family members induce the expression of PD-L1 [59,60,61]. Consequently, their baseline expression levels could be an indicator of PD-L1 manifestation in the tumor micro-environment, and thus help predict response to anti-PD-L1 immunotherapy [61]. According to recent developments, STAT1 emerged as a potential immunotherapy biomarker. Indeed, in their study, Zemek et al. compared the gene expression profiles of immune checkpoint inhibition responsive and non-responsive tumors in mice and validated their findings in cohorts of patients with cancer treated with immune checkpoint blocking antibodies [59]. They found that responsive tumors were characterized by an inflammatory gene expression signature consistent with an up-regulation of STAT1 signaling. This is particularly appealing in that their findings rendered possible the use of a biomarker-driven approach to patient management in order to properly establish whether a patient would benefit from treatment with sensitizing therapeutics before immune checkpoint blockade. In our study, the presence of a STAT2 protein with an abnormally elevated molecular mass combined to the presence of activated STAT3 distinguishes the T142 metastatic from the other non-metastatic UM cell lines and therefore militates toward a deeper involvement of both STAT2 and STAT3 in the aggressiveness of uveal melanoma. Analysis of the STAT2 and STAT3 expression and activation profiles in additional UM primary tumors and UM cell lines should prove particularly interesting to decipher whether both these mediators can be used as diagnostic markers for the identification of patients at risk of evolving toward liver metastatic disease.

In summary, we demonstrated that STAT proteins contribute to the transcription of the serotonin receptor HTR2B in uveal melanoma. The demonstration that STAT proteins can physically interact, or synergize with transcription factors such as NFI [35,36] that positively regulate transcription of the *HTR2B* gene, gives further support to the occurrence of an interleukin/JAK/STAT/NFI signalization cascade that likely contributes to the aggressiveness of uveal melanoma.

## 4. Materials and Methods

This study was conducted in agreement with the Helsinki declaration and was performed under the guidelines of the research ethics committee of the “CHU de Québec” (ethic code: F9-49776, protocol renewal approved on 4 September 2020).

### 4.1. Cell Culture

The UM cell lines T97, T98, T108, T111, T128, T131, T132, T142, T143, T151 and T157 were each cultured from the primary tumors of different patients diagnosed with this type of cancer and have already been previously described [33,62,63,64]. All cells were cultured in Dulbecco/Vogt modified Eagle’s minimal essential medium (DMEM) Multicell (high glucose, with l-glut, without L-Pyruvate; Wisent, Québec, QC, Canada) supplemented with 10% fetal bovine serum (FBS) High quality (Wisent, Québec, QC, Canada) and 0.002% *v*/*v* gentamicin (Life Technologies (distributed by Thermo Fisher Scientific Inc., Rockford, IL, USA) at 37 °C under 5% CO_2_.

### 4.2. Plasmid Construct, Oligonucleotides and Site-Directed Mutagenesis

Construction of the HTR2B/-2000 plasmid bearing the HTR2B promoter segment from −2000 to +96 relative to the theoretical mRNA start site has been described previously [32]. The putative STAT target site identified at position −280 upstream of the *HTR2B* theoretical mRNA start site was mutated into the HTR2B/-2000 construct (to yield HTR2B/-2000(MU_STAT-280_)) using the Quick Change Lightning Multi Site-Directed Mutagenesis Kit from Agilent Technologies (Santa Clara, CA, USA) according to manufacturer’s instructions (see Supplementary Table S1 for DNA sequence of the mutated primers used). The DNA insert from each recombinant plasmid was sequenced by chain-termination dideoxy sequencing (SANGER Sequencing platform, CHU de Québec-Université Laval Research Center, CHUL, Québec, Canada) to confirm the mutations.

The double-stranded oligonucleotides used either as labeled probe or unlabeled competitors in the EMSAs were chemically synthesized using a Biosearch 8700 apparatus (Integrated DNA Technologies, Inc., Coralville, WA, USA). Their DNA sequences are listed in Supplementary Table S1.

### 4.3. Transient Transfections and CAT Assays

The HTR2B/-2000 plasmids bearing the wild-type −280 STAT target site (HTR2B/-2000(WT_STAT-280_)) and its derivative bearing mutations in the −280 STAT site (HTR2B/-2000(MU_STAT-280_)) were transiently transfected into sub-confluent (80% coverage of the culture plate) T108 UM cells. Six-wells tissue-culture plates were used along with the K2^®^ Transfection System following the manufacturer’s instructions (BIONTEX, München, Germany). Each tissue-culture well received 1.5 g of the test plasmid and 0.5 g of the hGH-encoding plasmid PXGH5. All cells were harvested 48 h following transfection and CAT activities were determined and normalized to the hGH secreted in the medium as previously described [32]. Values shown are the mean of three separate transfections, each done in triplicate.

### 4.4. Preparation of Nuclear Extracts and EMSA

All tissue-cultured UM cells were grown to mid-confluence (70% coverage of the culture flasks) prior to preparation of nuclear extracts and their use in EMSAs following previously described procedures [65,66]. EMSAs were carried out by incubating 5 × 10^4^ cpm of the ^32^P-end-labelled, double-stranded oligonucleotide bearing the DNA sequence of the *HTR2B* −280 STAT site with the amount of nuclear proteins specified in the legend of each figure, in the presence of 2 μg of poly(dI:dC) (Amersham Biosciences, Piscataway, NJ, USA) and 50 mM KCl in buffer D [10 mM Hepes pH 7.9, 10% *v*/*v* glycerol, 0.1 mM EDTA, 0.5 mM DTT (dithiothreitol; Sigma-Aldrich Canada, Oakville, ON, Canada) and 0.25 mM phenylmethylsulfonyl fluoride (PMSF; Sigma-Aldrich Canada)]. DNA–protein complexes were next separated by gel electrophoresis through 8% non-denaturing polyacrylamide gels run against a Tris-glycine buffer (50 mM Tris, 2.5 mM EDTA, 0.4 M glycine) at 4 °C. The position of the DNA–protein complexes was then revealed upon autoradiography of the dried gels at −80 °C.

### 4.5. Western Blots

Western blots were conducted as described [32,62,63,64,67] using antibodies directed against the following proteins: Total STAT1 (D1K9Y (polyclonal), 1/300; Cell Signaling Technology), Total STAT2 (D9J7L (polyclonal), 1/300; Cell Signaling Technology, Danvers, MA, USA), Total STAT3 (sc-8019 (monoclonal), 1/100; Santa Cruz Biotechnology, Dallas, TX, USA), Total STAT4 (sc-398228 (monoclonal), 1/100; Santa Cruz Biotechnology), Total STAT5 (sc-74442 (monoclonal), 1/100; Santa Cruz Biotechnology), Total STAT6 (sc-374021 (monoclonal), 1/100; Santa Cruz Biotechnology), HTR2B (HPA-012867 (monoclonal), 1/100; Millipore Sigma), Phospho STAT1 (sc-8394 (monoclonal), 1/100; Santa Cruz Biotechnology), Phospho STAT3 (sc-8059 (monoclonal), 1/100; Santa Cruz Biotechnology), Phospho STAT5 (sc-81524 (monoclonal), 1/100; Santa Cruz Biotechnology), and a peroxidase-conjugated AffiniPure Goat secondary antibody against mouse IgG (1:2500 dilution; Jackson ImmunoResearch Laboratories, West Grove, PA, USA).

### 4.6. Gene Expression Profiling

Isolation of total RNA and microarray analysis, which all comply with the Minimum Information About a Microarray Experiment (MIAME) requirements, were conducted as recently reported [67]. All data generated from the arrays were also analyzed by robust multi-array analysis (RMA) for background correction of the raw values. They were then transformed in Log2 base and quantile normalized before a linear model was fitted to the normalized data to obtain an expression measure for each probe set on each array. Heat maps were generated using the ArrayStar V4.1 (DNASTAR, Madison, WI, USA) software. All microarray data presented in this study comply with the Minimum Information About a Microarray Experiment (MIAME) requirements. Data have been deposited in NCBIs Gene Expression Omnibus (GEO, available online: http://www.ncbi.nlm.nih.gov/geo/) and are accessible through GEO Series accession number GSE GSE86915 (last accessed date: 25 January 2022).

### 4.7. Statistical Analyses

A Student’s *t*-test was performed for comparison of the groups in transfection analyses. Differences were considered to be statistically significant at *p* < 0.05. All data are also expressed as mean ± SD.

## Data Availability

All microarray data presented in this study can be accessed at NCBI Gene Expression Omnibus (GEO# GSE86915; https://www.ncbi.nlm.nih.gov/geo/query/acc.cgi?acc=GSE86915) (last accessed date: 19 December 2022).

## Supplementary Materials

The following supporting information can be downloaded at: https://www.mdpi.com/article/10.3390/ijms23031564/s1.

## Abbreviations

EMSA	Electrophoretic mobility shift assay
