# Supplementary material for: Contribution of the STAT Family of Transcription Factors to the Expression of the Serotonin 2B (HTR2B) Receptor in Human Uveal Melanoma
Source: Int J Mol Sci. 2022 Jan 29;23(3):1564. doi: 10.3390/ijms23031564 (PMC8836204; doi:10.3390/ijms23031564)
Supplement: Supplementary file 1 [file ijms-23-01564-s001.zip › ijms-1541581-supplementary.pdf]

International Journal of
Molecular Sciences

MDPI

Supplementary materials

Contribution of the STAT family of transcription factors to the expression of the serotonin 2B (HTR2B) receptor in human uveal melanoma

Manel Benhassine [1], Gaëtan Le-Bel [1] and Sylvain L. Guérin [1,2,] *

[1] Centre Universitaire d'Ophtalmologie-Recherche (CUO-Recherche), Axe médecine régénératrice, Hôpital du Saint-Sacrement, Centre de Recherche FRQS du CHU de Québec, Université Laval, Québec, QC G1S4L8, Canada; manal.benhassine.1@ulaval.ca

[2] Département d'ophtalmologie, Faculté de Médecine, Université Laval, Québec, QC G1V0A6, Canada

* Correspondence: Sylvain.Guerin@fmed.ulaval.ca; Tel.: +1-(418)-682-7565

Supplementary Figures

A. STAT *DRE*: TTC $(N)_{2-4}$ GAA

-1862…TACT TTC TACT tAA AGTG…-1846
-1890…TACG TTC CTTA GAc ATTA… -1673
-1742…ATGC TTC CCA tAA CTTG… -1726
-1491…TTAT TTC ATA cAA GTCT… -1475
-1473…TCTT TTC ACT GgA TATA… -1457
-1409…TGCC TTC ATAA GcA CAGA… -1392
-1244…CAGG TTC ATG cAA TCCT… -1228
-602…CCAA TTt TCT GAA ATAA… -686
-559…TATT TTC TC cAA CGTA… -545
-244…ATTT TTC TC aAA TGTG… -229

B. STAT *DRE*: TTC $(N)_{2-4}$ GAA

-990…CATA TTC CT GAA TAGA… -975
-950…TGAT TTC TCTT GAA TATT… -933
-288…AATC TTC AAA GAA TGAA… -272

Supplementary Figure S1. Putative target sites for STAT proteins identified in the promoter and 5′-flanking sequence of the HTR2B gene. **(A)** Predicted sites (red sequences) that diverge from the consensus DRE by one base pair (identified in blue). **(B)** Putative sites that perfectly match the canonical STAT DRE. Numbers refer to the position of the sequences relative to the HTR2B putative mRNA start site. Thirteen STAT target sites could be identified along the entire promoter and 5′-flanking sequence of the HTR2B gene of which three (at positions -982, -942 and -280 relative to the HTR2B mRNA start site) perfectly match the STAT consensus sequence.

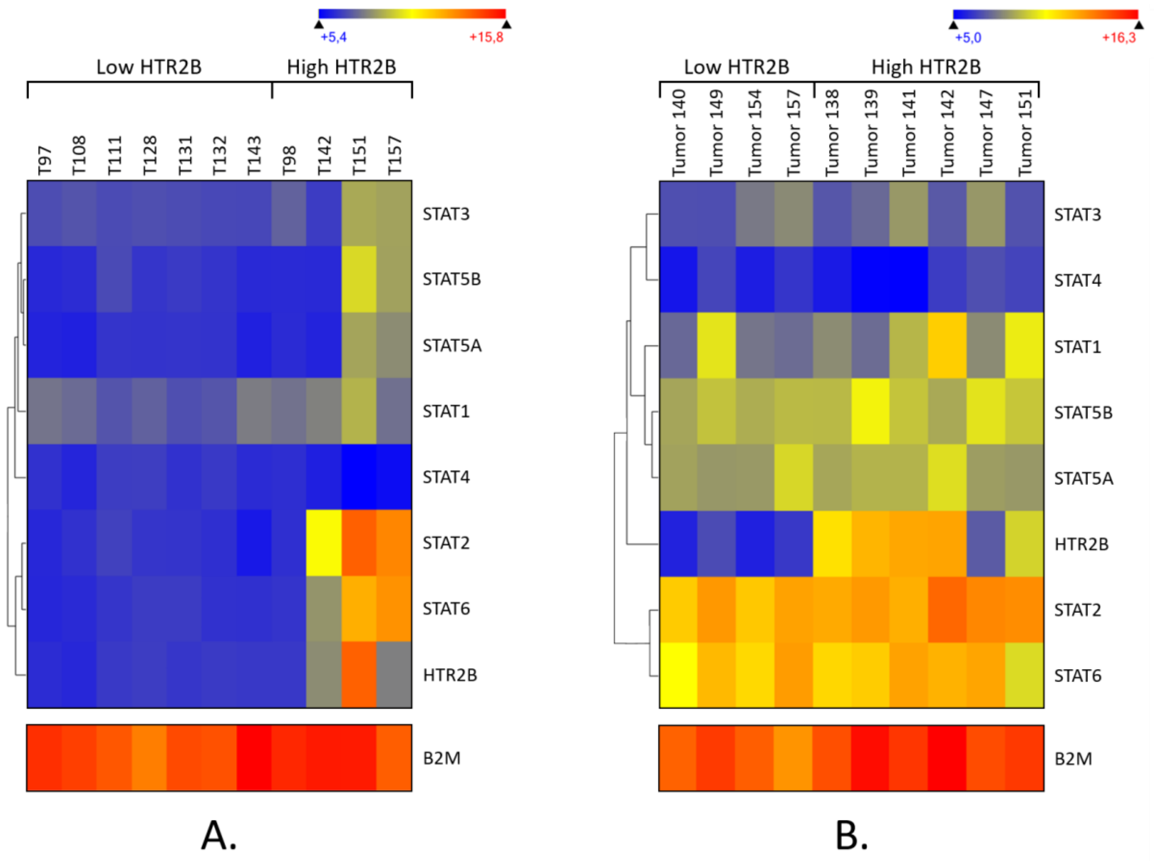

Supplementary Figure S2. Supplementary figure. Expression of STAT genes in uveal melanoma. **(A)** Heatmap representation of HTR2B and all the STAT genes expressed by UM cell lines. Data show the individual transcriptome profile extracted from UM cell lines that express either low (T97, T108, T111, T128, T131, T132 and T143 cell lines) or high levels of HTR2B (T98, T142, T151 and T157 cell lines). Moderate to high levels of STAT gene expression could only be observed in HTR2B-expressing UM cell lines. **(B)** Heatmap representation of HTR2B and all the STAT genes expressed by UM primary tumors. Data show the individual transcriptome profile extracted from UM primary tumors with either low (tumors 140, 149, 154 and 157) or high HTR2B levels (tumors138, 139, 141, 142, 147and 151). A dark blue color corresponds to a very low level of expression whereas high levels appear in yellow/red. Data are also presented for the housekeeping gene β-2-microglobulin (B2M; control).

Supplementary Tables

Supplementary Table S1. DNA sequence of synthetic oligonucleotides

Oligonucleotides used as labeled probes or competitors in the EMSAs

Oligonucleotide	Top strand (5′-3′)<br>Bottom strand (5′-3′)
**AP-1**	GATCCCCGCGTTGAGTCATTCGCCTC<br>GATCGAGGCGAATGACTCAACGCGGG
**NFI**	TTATTTTGGATTGAAGCCAATATGAG<br>CTCATATTGGCTTCAATCCAAAATAA
**Sp1**	GATCATATCTGCGGGGCGGGGCAGACACAG<br>GATCCTGTGTCTGCCCCGCCCCGCAGATAT
**STAT -280**	GATCGGCAGAATCTTCAAAGAATGAAACC<br>GATCGGTTTCATTCTTTGAAGATTCTGCC
**STAT 280 mutant**	GATCGGCAGAATCAACAAATAATGAAACC<br>GATCGGTTTCATTATTTGTTGATTCTGCC

Oligonucleotides used for site-directed mutagenesis

WT Sequence

5′ GGC TTT TAA AAC GGC AGA ATC **TT**C AAA **G**AA TGA AAC CTC TAA AAT AGC 3′

Mutated STAT putative binding Site

5′ GGC TTT TAA AAC GGC AGA ATC **AA**C AAA **T**AA TGA AAC CTC TAA AAT AGC 3′
